# Effects of Flaxseed on Perimenopausal Symptoms: Findings From a Single-Blind, Randomized, Placebo-Controlled Study

**DOI:** 10.7759/cureus.68534

**Published:** 2024-09-03

**Authors:** Rashmi Shrivastava, Sandeep Bhattacharya, Narsingh Verma, Abbas A Mehdi, Amita Pandey, Jamal A Ansari

**Affiliations:** 1 Physiology, King Georges Medical University, Lucknow, IND; 2 Physiology, Hindi Institute of Medical Sciences, Lucknow, IND; 3 Biochemistry, Era's Lucknow Medical College and Hospital, Lucknow, IND; 4 Obstetrics and Gynaecology, King Georges Medical University, Lucknow, IND; 5 Clinical Biochemistry, Integral University Lucknow, Lucknow, IND

**Keywords:** lignan, perimenopausal symptoms, metabolism, enterolactone, enterodiol

## Abstract

Background

Flaxseed contains many phytoestrogens that share structural similarities with endogenous estrogens. It has beneficial properties that improve hormonal effects that may influence chronic disorders.

Objective

This study aims to evaluate the impact of flaxseed supplementation on perimenopausal syndrome symptoms and serum polyphenol metabolites enterodiol (ED) and enterolactone (EL).

Methods

Recruitment of 145 perimenopausal women from Queen Mary's Hospital OPD in K.G.M.U. Lucknow, India, was done for this single-blind randomized placebo-controlled experiment, considering inclusion and exclusion criteria. We randomly divided subjects into two groups using a computer-generated random number table. The intervention group A received 10 grams of flaxseed powder, while the placebo group B received 10 grams of roasted wheat flour for a continuous three months. We measured their demographic characteristics, menopausal symptoms score using the modified Kupperman's index (KI), the menopause rating scale (MRS), and the menopause-specific quality of life (MENQOL) intervention questionnaire. Moreover, using high-performance liquid chromatography (HPLC), biochemical parameters (ED, EL) were assessed both at baseline and after three months. For the statistical analysis, Statistical Product and Service Solutions (SPSS, version 24; IBM SPSS Statistics for Windows, Armonk, NY) was employed.

Result

The study involved 123 individuals, with 22 subjects losing out during follow-up. From baseline to follow-up comparison, the intervention group demonstrated an average percentage decline in the final score of the KI, MRS, and MENQOL intervention questionnaire of -47.25%, -54.05%, and -50.39%, respectively, while the placebo group demonstrated an average percentage decline of -3.83%, -4.91%, and -7.92%, respectively. From baseline to follow-up, ED and EL levels increased by 2.81-fold and 8.55-fold in the intervention group and decreased by 0.25-fold and 0.27-fold in the placebo group.

Conclusion

Following three months of supplementation, the intervention group showed substantially lower menopausal symptoms (p < 0.001), and ED and EL levels were considerably higher (p < 0.001). Therefore, flaxseed might ameliorate the symptoms associated with perimenopause.

## Introduction

Perimenopause is the time frame right before and up to a year after the last menstrual period in the Clinical Practice Guidelines on Menopause (Indian Menopause Society, 2019-2020). It could take three or five years. Ovarian follicular function loss results in a drop in progesterone and estrogen levels in the ovaries; the World Health Organization (WHO) describes perimenopause as the permanent end of menstruation.

Many Indian women under 45 years of age may experience a marked rise in the prevalence of perimenopausal syndrome during the next few decades. In India, many studies have reported a mean age at menopause ranging from 41.9 to 49.42 [1]. Globally, it was discovered that approximately 48.8 years was the average age at natural menopause [2]. Numerous symptoms have been linked to menopause. These include vasomotor symptoms such as night sweats, hot flashes, and irregular vaginal flow; psychological symptoms such as anxiety, depression, crying fits, irritability, and poor sleep; and physical symptoms such as headaches, body aches, and joint problems [3].

The only approved treatment for perimenopausal syndrome is hormone therapy. We know that hormone therapy has some side effects, particularly cancer risk, so there is a need for complementary and alternative therapy. Flaxseed is by far the most abundant source of lignan. It has gained attention in the fields of nutrition and disease due to its physiologically active components. Secoisolariciresinol (SECO) is the major lignan present in flaxseed, which is found as the conjugate diglycoside, secoisolariciresinol diglucoside (SDG) [4]. In the colons of humans and other animals, microorganisms transform plant lignan, known as SDG, into the mammalian lignans enterodiol (ED) and enterolactone (EL). EL, a mammalian lignan, can be produced by the intestinal microbiota by the oxidation of ED. Plants and plant-derived products include diphenolic compounds, sometimes referred to as dietary estrogens or phytoestrogens, that have some estrogenic or antiestrogenic qualities. Lignans are also a type of phytoestrogen with a structure such as that of endogenous sexual steroid hormones. It has received very little attention despite being present in more naturally occurring foods and being consumed more frequently by populations [4,5].

Many researchers have examined how different diets or dietary components affect the secretion of lignan in a range of groups, including women who are premenopausal, menopausal, and postmenopausal. However, the area of perimenopause remains unexplored. To our knowledge, a scarcity of literature exists on the impact of flaxseed administration on lignan metabolites in individuals with perimenopausal syndrome. Lignan metabolites (e.g., ED and EL) have weak estrogenic properties, so they may help in the amelioration of symptoms of perimenopausal syndrome. Therefore, this study focuses on the objective of examining how perimenopausal women's blood levels of lignan metabolites and associated symptoms are affected when they take flaxseed supplements.

## Materials and methods

Study design

A total of 250 participants were enrolled in this study after being referred by a medical professional in the outpatient department (OPD) of Queen Mary's Hospital of Obstetrics and Gynecology Department, K.G.M.U., Lucknow. Overall, 200 subjects were screened in this single-blind, randomized, placebo-controlled study after 50 participants initially refused to participate in the study. According to the inclusion and exclusion criteria, 145 recruited patients were randomly assigned into two groups: intervention group A (n=72) and placebo group B (n=73), using a random number table generated by a computer. Thirty people were excluded for not meeting the inclusion criteria; 13 people declined to participate, and 12 were excluded because of other reasons. Following the assignment of subjects to the groups, baseline parameters (e.g., clinical, biochemical, anthropometric, and demographic) were evaluated before and upon completion of the three-month follow-up. After finishing their respective supplements on completion of a three-month follow-up, 63 and 60 participants in the intervention and placebo groups, respectively, based on the group they were assigned, finished the trial. The loss to follow-up was nine in the intervention group and 13 in the placebo group. The current study was registered with the Clinical Trial Registry of India (CTRI/2022/09/045980) and authorized by the Institutional Ethics Committee of K.G.M.U., Lucknow (Ref Code: 110th EMC II B-PhD/P5).

Subjects

**Figure 1:**
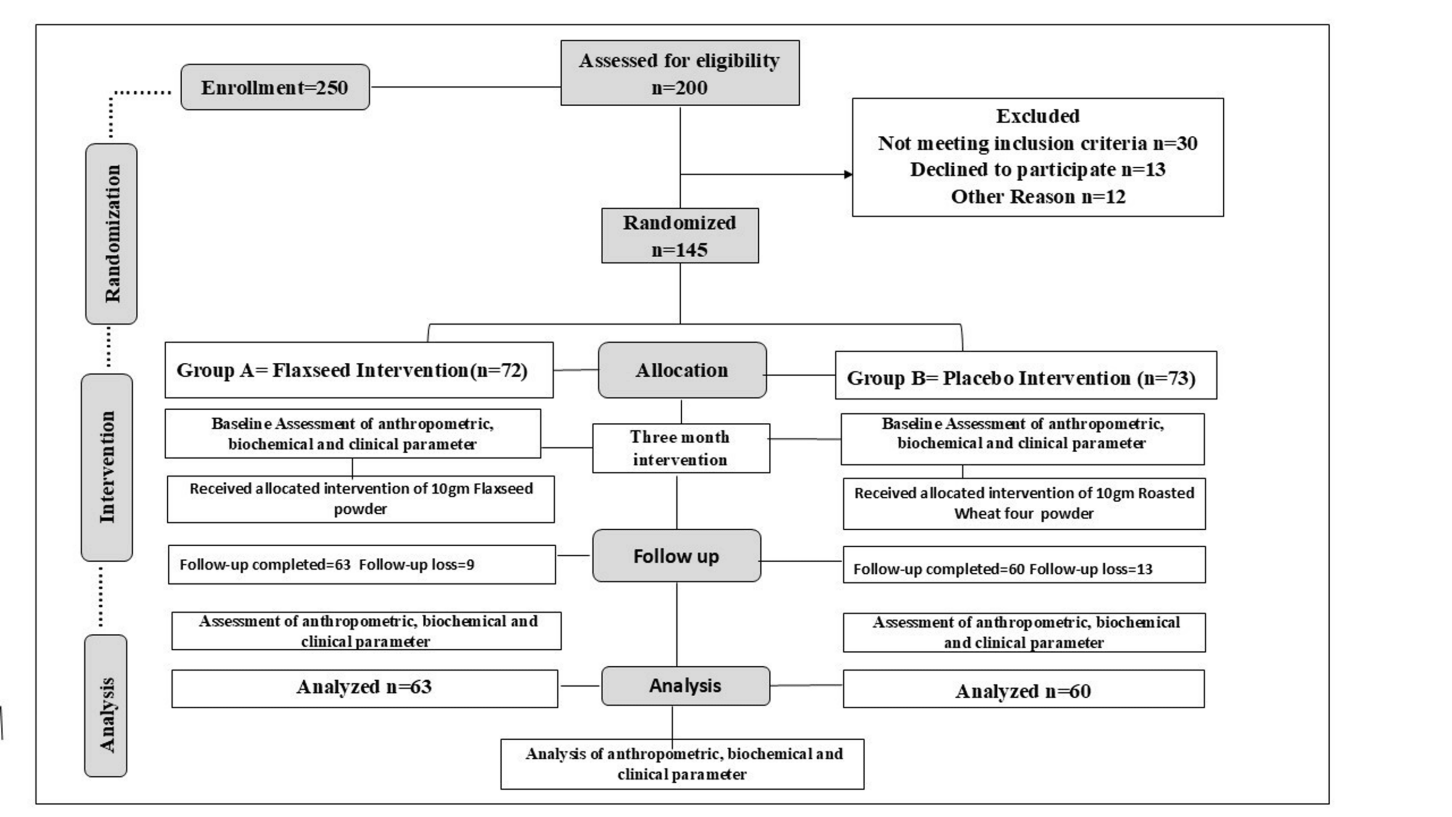
Study flowchart

As per the inclusion criteria, subjects aged 40-55 years were enlisted under the International Classification of Diseases (ICD-10) N-95.9 (unspecified menopausal and perimenopausal disorder) [6]. Subjects who did not take any additional supplements or foods (e.g., soybean, chia seed, and so on) that contained a high number of flavonoids or isoflavonoids at the time of study or at least three months before the study and who were not on pharmaceutical conventional treatment (estrogen and phytoestrogen) were also included. Those who had undergone surgical menopause, hysterectomy, abnormal uterine or ovarian anatomy, hormone replacement therapy (HRT), selective serotonin reuptake inhibitors (SSRIs), mental disorders (including depression), or thyroid diseases were not eligible to participate in this study. Moreover, patients with cardiovascular, hepatic, or renal disorders, those taking sedative or anti-anxiety medications, and those who abused narcotics or cigarettes were also excluded from the study. In Figure 1, the study flowchart is displayed.

Intervention and placebo group

In the intervention group, 10 gm of flaxseed powder in an amber glass bottle with a code labeled at the base was provided to participants while in the placebo group, the same amber glass bottle containing roasted wheat flour had a different code at the base that was only understandable by the researcher, not the subjects, to preserve single blindness in the study was provided. As stated, in the single-blinded study, only patients were blinded to the given interventions. However, the researcher and investigators were aware of it. Storage of the supplements in a cool, dry place was advised for the individuals. Furthermore, the participants were directed to consume one bottle of supplement in a whole day. The whole wheat flour and flaxseed powder were provided by a commercial provider, Ceyon Healthcare Private Limited. Compliance was monitored with regular emails, weekly phone conversations, and monthly bottle collection. The research participants were instructed to maintain their usual eating and exercise regimen.

Anthropometric parameter assessment

An analog weighing scale and a vertical stadiometer were used to measure the participant's height and weight. Utilizing these values, the BMI was computed and categorized by WHO standards. According to WHO classification, waist circumference (WC) is measured at the midpoint between the iliac crest and lower ribs using an inelastic and inextensible measuring tape [7,8].

Biochemical investigations

Sample Collection and Analysis

To assess the biochemical parameters (ED, EL level), 12 hours following their last meal, in the morning OPD, blood samples were taken from both groups using a standard vacutainer. After separation through centrifugation at 3,000 rpm for 15 minutes, the serum was refrigerated at -20°C until examination. ED and EL's biochemical characteristics were examined using high-performance liquid chromatography (HPLC) with the coulometric array detection method. The sample processing methodology was based on Paul H. Gamache and Ian N. Acworth’s 1998 work [9]. The Shimadzu Prominence instrument (model no. L202256) with pump LC-20AD and detector SPDM20A diode array detector was used for the analysis. The C-18 (4.6 x 250 mm) 5-um pore size column was utilized in this investigation. A V190 series computer running Lab Solutions software was used to manage the system to gather, process, and store data. The correlation coefficient of least squares linear regression was employed to gauge linearity.

Clinical Symptoms Assessment

Three instruments were used to quantify clinical symptoms: Modified Kupperman’s index (KI) [10], the menopause rating scale (MRS) [11], and the menopause-specific quality of life (MENQOL) intervention questionnaire [12].

There are 13 items in the modified KI. The complaints' severity was rated on a zero to three-point scale. After adding together each element in the weighting factor, a total score between zero and 63 is produced. There were four different ways to grade the level of severity: none, mild, moderate, and severe. The scores went from 0-6, 7-15, 16-30, and >30.

The MRS consists of eleven items, with scores ranging from zero (no complaints) to four (very severe symptoms). Points are given for each subjectively assessed level of symptom intensity. By checking one of five "severity" boxes, the respondent expressed how seriously they took each item.

The MENQOL intervention questionnaire is a 32-item intervention. Four domains were identified from these questions: vasomotor (three items), sexual (three items), physical (19 items), and psychosocial (seven items). The subjects were asked to score the severity of their symptoms on a seven-point Likert scale from zero to six. If “no,” the subject goes to the next item; however, if “yes,” she indicates how bothered she was by the item on a seven-point Likert scale ranging from “0”: “not at all bothered” to “6”: “extremely bothered.” For analysis, the questionnaire score becomes “1” for “no,” “2” for “yes,” “not bothered” through to “8” for “yes,” and “extremely bothered.” The score by domain is the mean of the converted item scores forming that domain and ranges from 1 to 8. The domain score's mean was the overall MENQOL score. Tools for this study were translated into Hindi, the native language by the psychologist and statistician. The factor loading scale covered the interval from -1.0 to +1.0. Cronbach's Alpha was used to assess the tool's reliability, and the test value ranges from 0.60 to 0.80.

Statistical Analysis

In this study, data were presented as number (n), mean, SD, and percentage (%). The Kolmogorov-Smirnov test was performed to determine whether the dataset followed a normal distribution or not. After checking for normality, for the within-group comparison, a paired t-test was used for parametric data, and a Wilcoxon-matched pair test was used for nonparametric data. For between-group comparison, an independent t-test was used for parametric data, and the Mann-Whitney U test was used for nonparametric data. Significant P values were defined as those less than 0.05, 0.01, 0.001 is significant, moderately significant, and highly significant, respectively. The effect size was calculated as the average difference between the mean of intervention and roasted wheat flour placebo divided by the SD of the roasted wheat flour placebo differences [13]. Statistical Product and Service Solutions (SPSS, version 24; IBM SPSS Statistics for Windows, Armonk, NY) software was used for all statistical analyses that we conducted in this study.

## Results

The intervention group and placebo group had mean ages of 45.44±3.59 and 45.41±3.27, respectively. The anthropometric factors (e.g., weight, height, BMI, and waist circumference) and the demographic variables (e.g., age, marital status, and parity) were not found to be statistically significant when compared between both groups at baseline. In terms of marital status, 95.2% of subjects were married in the intervention group, while 91.7% of subjects were married in the placebo group. In the other three categories, 1.6% of patients were unmarried, 1.6% were widows, and 1.6% were divorced in the intervention group. Approximately 8.3% of individuals were widows in the placebo group.

Baseline data indicate the presence of grade 1 obesity in subjects based on BMI and increased waist circumference [7] in both the intervention and placebo groups. The demographic and anthropometric data at baseline in both groups are shown in Table 1.

**Table 1:** Demographic and anthropometric data at baseline in two groups Values are expressed in mean ± SD; u: Mann–Whitney U test; ^ns^p>0.05

S.N.	Demographic and anthropometric parameters		Intervention (n=63)	Placebo (n=60)	P value
1	Age (years) (m±SD)		45.44±3.59	45.41±3.27	^u^p^ns^=0.957
2	Marital Status n (%)	Unmarried	1 (1.6%)	0	NA
		Married	60 (95.2%)	55 (91.7%)	
		Widow	1 (1.6%)	5 (8.3%)	
		Divorced	1 (1.6%)	0	
3	Parity n (%)	0	1 (1.6%)	0	NA
		1	5 (7.9%)	8(13.3%)	
		2	30 (47.6%)	26(43.3%)	
		≥3	27 (42.9%)	26(43.3%)	
4	Weight (kg) (m±SD)		65.62±11.89	64.46±12.71	^u^p^ns^=0.509
5	Height (cm) (m±SD)		152.21±6.08	152.21±6.03	^u^p^ns^=0.913
6	BMI (m±SD)		28.29±4.83	27.74±4.74	^u^p^ns^=0.410
7	Waist circumference (cm) (m±SD)		87.17±8.69	85.68±10.50	^t^i^ns^=0.392

Variations in anthropometric characteristics from baseline to follow-up after three months of intervention

There is more reduction in the mean±SD value of BMI (i.e., 28.29±4.83 to 28.17±4.71 in the intervention), compared to placebo 27.74±4.74 to 27.72±4.68. In the case of the waist circumference, the intervention group showed more reduction in the mean±SD value as 87.17±8.69 to 86.77±8.58, in comparison with the placebo (i.e., 85.68±10.50 to 85.45±10.36). When comparing the variable from baseline to follow-up, the intervention group showed a substantial decrease in both the BMI and waist circumference (p<0.01, p<0.05, respectively). However, the placebo group exhibited negligible change (Table 2).

**Table 2:** Variations in anthropometric parameters between the groups and from baseline to three-month follow-up within the group Values are expressed in mean±SD; w=Wilcoxon matched pair test; t=paired t-test; u=Mann–Whitney U test; nsp>0.05; *p<0.05; **p<0.01.

S.N.	Variable		Baseline (m±SD)	Three-month follow-up (m±SD)	% change	P value	
						Within group at baseline up to three-month follow-up	Between groups at baseline
1	BMI	Intervention (n=63)	28.29±4.83	28.17±4.71	0.42	^w^p**=0.001	^u^p^ns^=0.410
		Placebo(n=60)	27.74±4.74	27.72±4.68	0.07	^t^p^ns^=0.620	
2	Waist circumference (cm)	Intervention (n=63)	87.17±8.69	86.77±8.58	0.45	^t^p^*^=0.037	^t^p^ns^=0.392
		Placebo (n=60)	85.68±10.50	85.45±10.36	0.26	^t^p^ns^=0.341	

Variation in modified KI scores from baseline to follow-up after three months of intervention

The mean score of the clinical variables (subscales and total score) of the modified KI was not significant statistically (p > 0.05) when comparing the two groups at baseline. The somatic subscale scores in the intervention group decreased from 27.22±5.36 to 17.34±3.39, the psychological scores decreased from 8.38±2.41 to 3.17±2.09, the urogenital scores decreased from 7.69±3.25 to 2.31±2.14, and the total score decreased from 43.30±8.42 to 22.84±5.49. The subscale scores in the placebo group, however, decreased less. These subscale scores were somatic (25.76±4.61 to 25.11±5.05), psychological (7.80±2.46 to 7.28±2.23), urogenital (7.66±5.95 to 7.25±2.72), and total (41.23±6.19 to 39.65±6.62). The intervention group showed changes in subscale scores in percentages of (-47.25%, -36.29%, -62.17%, -69.96%), in comparison to the placebo group (-3.83%, -2.52%, -6.66%, -5.35%). Except for the somatic and urogenital subscales with an effect size of 0.31 and 0.23, respectively, and with p<0.001 (within the group) showed a high statistically significant difference in the intervention group compared to the placebo, there was an overall less statistically significant difference between the two groups among other variables.

Variation in MRS scores from baseline to follow-up after three months of intervention

When comparing the two groups at their baseline, the mean score of the MRS' clinical factors (subscales and total score), there was no statistical significance (i.e., p>0.05). The mean and SD of scores of subscales were decreased from the baseline to follow-up, in the intervention group (i.e., in the somatic subscale (11.14±2.55 to 5.28±2.31), psychological (9.74±3.04 to 4.09±2.84), genitourinary (6.88±2.38 to 3.38±1.67) and in the total score (27.77±5.79 to 12.76±5.22)). However, the subscale scores (somatic subscale (10.50±2.49 to 9.86±2.07), psychological (8.95±3.26 to 8.51±3.51), genitourinary (6.58±2.47 to 6.38±2.15), total score (26.03±6.37 to 24.75±6.39)) were reduced less in the placebo group. The percentage changes in subscale scores between the intervention and placebo groups were (-54.05%, -52.60%, -58%, -50.87%) and (-4.91%, -6.09%, -5.02%, -3.03%), respectively. Without exception, the genitourinary subscale (with an effect size of 0.12) demonstrated a high statistically significant difference (p<0.001), while other variables showed an overall lesser statistically significant difference. 

Variation in scores of the MENQOL intervention questionnaire from baseline to follow-up after three months of intervention

**Figure 2:**
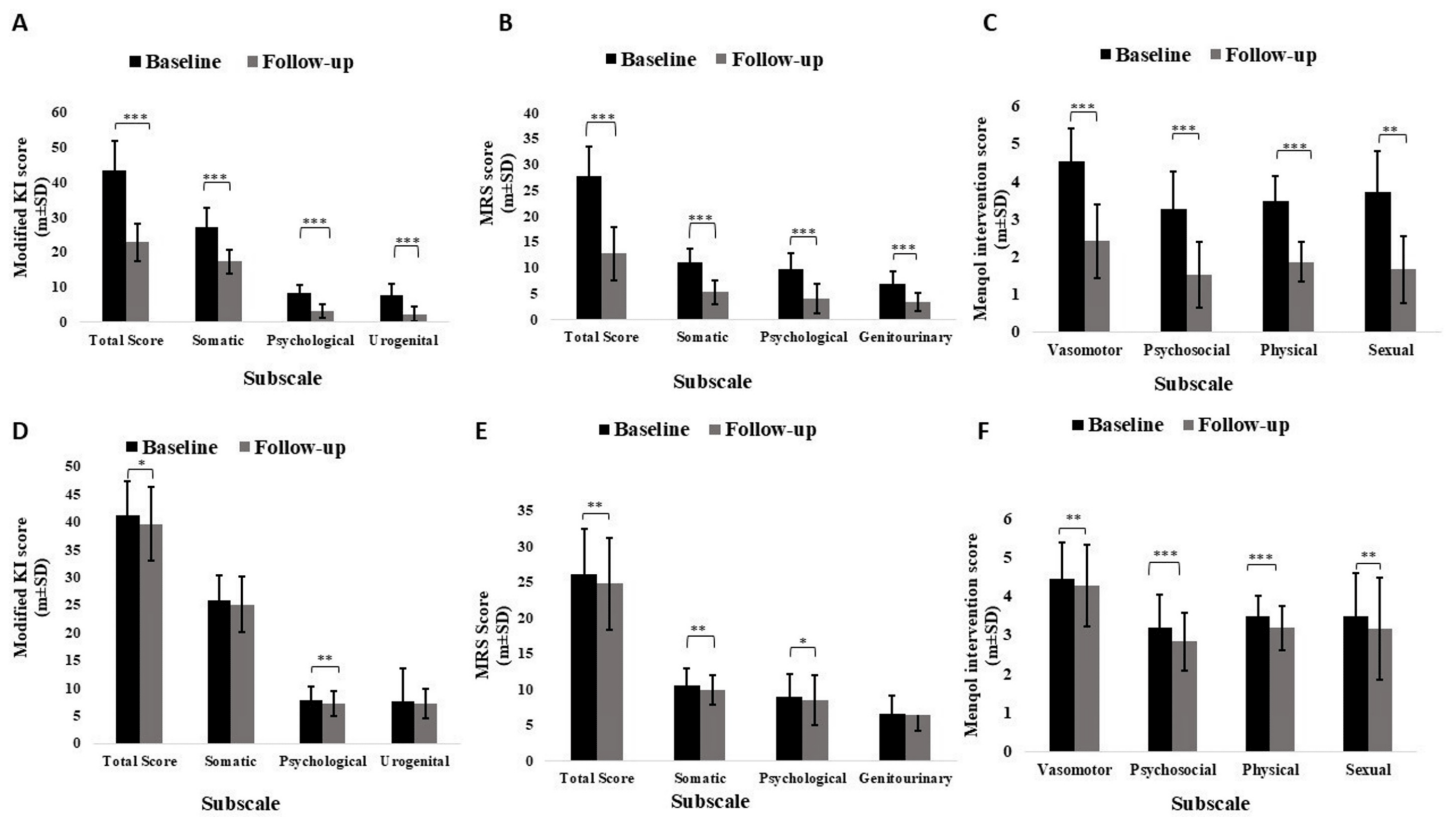
Clinical characteristics: changes in Modified Kupperman index score, MRS, menopause-specific quality of life questionnaire of the intervention group (A, B, C), and placebo group (D, E, F) at their baseline, as well as from baseline to follow-up between the groups Data are expressed in mean±SD; level of significance p<0.001=***; p<0.01=**; p<0.05=*; n=number of subjects; m=mean; modified KI=modified Kupperman index; MRS=menopause rating scale; MENQOL=menopause-specific quality of life questionnaire.

At baseline, there was no statistically significant difference in the (subscale) mean score and the summary score of the MENQOL intervention questionnaire. The mean and SD of scores of subscales have decreased from the baseline to follow-up, in the intervention group (i.e., in the vasomotor subscale): 4.54±0.89 to 2.42±0.99, psychosocial (3.29±0.99 to 1.53±0.88), physical (3.50±0.66 to 1.87±0.52), sexual (3.73±1.10 to 1.67±0.89) and summary score (3.77±0.71 to 1.87±0.66). In the placebo group, there was less reduction in the subscale scores (i.e., in vasomotor subscale (4.46±0.93 to 4.28±1.05), psychosocial (3.20±0.85 to 2.84±0.75), physical (3.49±0.53 to 3.19±0.58), sexual (3.50±1.09 to 3.17±1.31), and the summary score (3.66±0.65 to 3.37±0.68). In the intervention group, changes in subscale scores given as percentages were (-46.69%, -53.49%, -46.57%, -55.22%, -50.39%), while in the placebo group, changes in subscale scores as percentages were (-4.03%, -11.25%, -8.59%, -9.42%, -7.92%). These changes showed overall less statistical significance among both the groups (Figure 2).​​​​​​​

Variation in biochemical parameters from baseline to follow-up after three months of intervention

**Figure 3:**
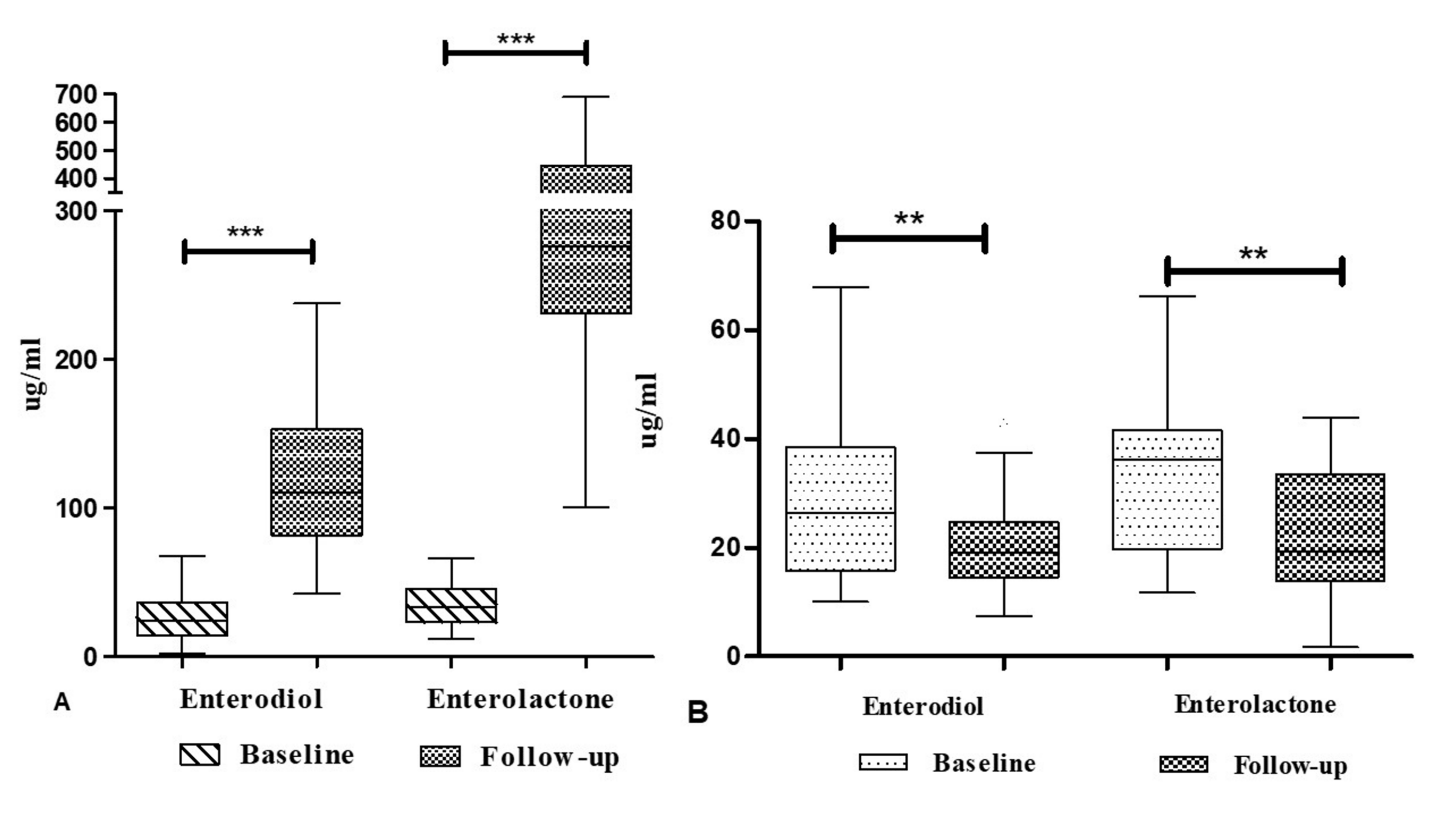
Enterolignan metabolite levels at their baseline and from baseline to follow-up in the intervention (A) and placebo (B) groups Data are expressed in mean±SD; level of significance p<0.001=***; p<0.01=**; n=number of subjects; M=mean; ug=microgram; mL=milliliter

When baseline values of biochemical parameters were compared, overall no statistical difference was seen. The levels of lignan metabolites (ED and EL) in the intervention group grew significantly from baseline to follow-up (+2.81-fold, +8.55-fold) (p < 0.001), while the levels in the placebo group dramatically fell (-0.25-fold, 0.27-fold) (p < 0.01) (Figure 3). 

## Discussion

Most people in India from lower socioeconomic backgrounds, and low- and even middle-class populations find it extremely difficult to afford the HRT required to treat perimenopausal syndrome. Considering the negative impacts or long-term side effects of use, accessible and healthy alternatives are urgently needed to improve middle-aged women's quality of life. The effectiveness of administering ground flaxseed powder was assessed in this randomized, single-blind, placebo-controlled research by comparing it with a placebo. Additionally, menopausal symptoms and diet-derived polyphenol metabolite levels were assessed at baseline and three months into the follow-up period. Furthermore, the study included three separate instruments to assess symptoms, incorporating additional factors such as the application of instruments relevant to the Indian scenario and tools tailored to the intervention for analysis. It also examines the link between lignan metabolites following analysis and provides background information.

In a study, while retaining the same body weight and BMI, adults who ingested 100 mg/day of SDG, which was equivalent to 7.5 gm of flaxseed, reported a significant reduction in their WC [14]. Taking 40 gm/day of ground golden or brown flaxseed may have reduced the incidence of metabolic problems in perimenopausal overweight women with decreased WC [15]. Our results align with that study as the intervention group's percentage change for BMI and waist circumference (-0.42% and -0.45%, respectively) were slightly lower than those of the placebo group (-0.07% and -0.26%, respectively). The bioactive component of flaxseed could account for this variation, but it is also possible that the people's grade 1 obesity and their compliance with the semi-controlled diet played a role.

When perimenopausal women are given linseed (*Linum usitatissimum*), most of their climacteric phase symptoms are successfully reduced, according to a study showing the effects of a flaxseed diet on menopausal symptoms. In a study based on the research index [16], some other research examining the impact of flaxseed showed a decrease in menopausal symptoms (Kupperman-Blatt) [17,18]. Our results are consistent with earlier research as the intervention group’s KI subscale score decreased more than the placebo group, and both the somatic and urogenital subscales were the only areas where the groups' differences were statistically significant. In the MRS scale also, the intervention group showed a greater decrease in subscale scores, and in the genitourinary subscale, the difference within the group was statistically significant. The same result was also shown in the MENQOL intervention questionnaire, with a greater decrease in symptom scores in the intervention group compared to placebo, but the difference was overall less statistically significant. According to the MRS and SF-36 quality of life scale, menopausal symptoms were dramatically reduced in the experimental groups in a study that gave 5 mg daily for 12 weeks during the menopausal period [19]. Following the consumption of the mix, which included flaxseed, research participants in various studies showed a noteworthy amelioration in their somatic, urogenital, and psychological problems associated with menopause. Hot flashes, nocturnal sweats, constipation, and flatulence, as well as psychological problems including restlessness, worry, and difficulties falling asleep, all disappeared for the women [20]. The rise in plant-based estrogens explains the amelioration of vasomotor symptoms [21] as it is supposed that the hypothalamus regulates temperature homeostasis through norepinephrine and serotonin and in addition to the reduced gonadal hormone levels during the climacteric, it may cause instability in the central nervous system's noradrenaline and serotonin concentrations, altering the organism's thermoregulation and increasing the frequency of heat waves. After doing an intragroup study, [22] discovered a decrease in the psychological symptoms, particularly mood swings brought on by hypoestrogenism [23], which is typical for women throughout this stage of their life. Women who took 1,000 mg of flaxseed daily for six weeks saw a decrease in the length and frequency of menopausal symptoms, such as hot flashes and night sweats [24]. This result is comparable to that of a study [25], which showed a 3.3-fold increase after supplementing with 25 mg/day of linseeds [26]. According to our investigation, adding 10 gm of flaxseed powder to the diet raised ED levels by 2.81-fold (p < 0.001), and EL levels by 8.55-fold (p < 0.001) in the intervention group while in the placebo group, levels of lignan decreased by 0.25-fold (p < 0.01), 0.27-fold (p < 0.01), respectively.

Our study showed the beneficial effects of flaxseed intervention on perimenopausal patients after three months of supplementation. However, this single-blind study has a few limitations as it involves the findings observed for the three menstrual cycles (i.e., three months). Additionally, for more consistent results, in future studies, a period of six months can also be considered. Moreover, we have studied a small sample size, so for more generalizability of the findings, a larger sample size can be considered before planning future clinical trials and for the development of quality-of-life programs for perimenopausal patients.

## Conclusions

Our research indicates that 10 gm of flaxseed powder significantly enhanced EL and ED secretion and may be useful in easing perimenopausal symptoms. To confirm and build on the promising results of our study, additional investigation is necessary, including testing other doses and evaluating additional neuroendocrine, metabolic, and related gene expression markers with perimenopausal syndrome. Therefore, a novel strategy for treating perimenopausal syndrome patients without side effects will be developed, improving their quality of life.
